# Acetylcholinesterase: A Versatile Template to Coin Potent Modulators of Multiple Therapeutic Targets

**DOI:** 10.1021/acs.accounts.3c00617

**Published:** 2024-02-09

**Authors:** F. Javier Luque, Diego Muñoz-Torrero

**Affiliations:** † Department of Nutrition, Food Science and Gastronomy, Faculty of Pharmacy and Food Sciences, E-08921 Santa Coloma de Gramenet, Spain; ‡ Institute of Biomedicine (IBUB), University of Barcelona, E-08028 Barcelona, Spain; § Institute of Theoretical and Computational Chemistry (IQTC), University of Barcelona, E-08028 Barcelona, Spain; ∥ Laboratory of Medicinal Chemistry (CSIC Associated Unit), Faculty of Pharmacy and Food Sciences, University of Barcelona, E-08028 Barcelona, Spain

## Abstract

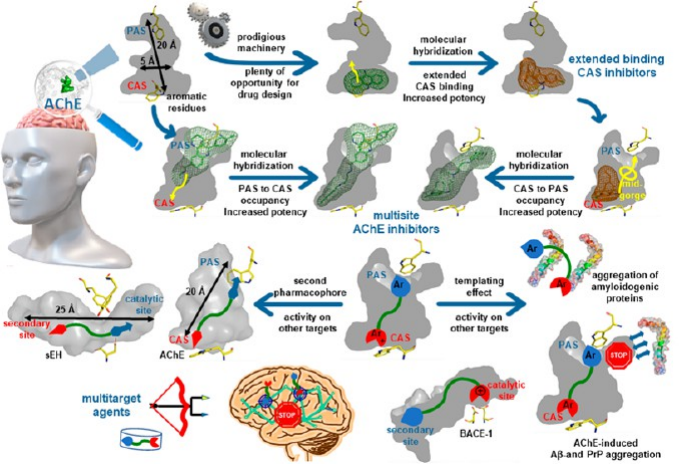

The enzyme acetylcholinesterase (AChE) hydrolyzes the neurotransmitter acetylcholine (ACh) at cholinergic synapses of the peripheral and central nervous system. Thus, it is a prime therapeutic target for diseases that occur with a cholinergic deficit, prominently Alzheimer’s disease (AD). Working at a rate near the diffusion limit, it is considered one of nature’s most efficient enzymes. This is particularly meritorious considering that its catalytic site is buried at the bottom of a 20-Å-deep cavity, which is preceded by a bottleneck with a diameter shorter than that of the trimethylammonium group of ACh, which has to transit through it. Not only the particular architecture and amino acid composition of its active site gorge enable AChE to largely overcome this potential drawback, but it also offers plenty of possibilities for the design of novel inhibitor drug candidates.

In this Account, we summarize our different approaches to colonize the vast territory of the AChE gorge in the pursuit of increased occupancy and hence of inhibitors with increased affinity. We pioneered the use of molecular hybridization to design inhibitors with extended binding at the CAS, reaching affinities among the highest reported so far. Further application of molecular hybridization to grow CAS extended binders by attaching a PAS-binding moiety through suitable linkers led to multisite inhibitors that span the whole length of the gorge, reaching the PAS and even interacting with midgorge residues. We show that multisite AChE inhibitors can also be successfully designed the other way around, by starting with an optimized PAS binder and then colonizing the gorge and CAS. Molecular hybridization from a multicomponent reaction-derived PAS binder afforded a single-digit picomolar multisite AChE inhibitor with more than 1.5 million-fold increased potency relative to the initial hit. This illustrates the powerful alliance between molecular hybridization and gorge occupancy for designing potent AChE inhibitors.

Beyond AChE, we show that the stereoelectronic requirements imposed by the AChE gorge for multisite binding have a templating effect that leads to compounds that are active in other key biological targets in AD and other neurological and non-neurological diseases, such as BACE-1 and the aggregation of amyloidogenic proteins (β-amyloid, tau, α-synuclein, prion protein, transthyretin, and human islet amyloid polypeptide). The use of known pharmacophores for other targets as the PAS-binding motif enables the rational design of multitarget agents with multisite binding within AChE and activity against a variety of targets or pathological events, such as oxidative stress and the neuroinflammation-modulating enzyme soluble epoxide hydrolase, among others.

We hope that our results can contribute to the development of drug candidates that can modify the course of neurodegeneration and may inspire future works that exploit the power of molecular hybridization in other proteins featuring large cavities.

## Key References

- CodonyS.; PontC.; Griñán-FerréC.; Di Pede-MattatelliA.; Calvó-TusellC.; FeixasF.; OsunaS.; Jarné-FerrerJ.; NaldiM.; BartoliniM.; LozaM. I.; BreaJ.; PérezB.; BartraC.; SanfeliuC.; Juárez-JiménezJ.; MorisseauC.; HammockB. D.; PallàsM.; VázquezS.; Muñoz-TorreroD.Discovery and In Vivo Proof of Concept of a Highly Potent Dual Inhibitor of Soluble Epoxide Hydrolase and Acetylcholinesterase for the Treatment of Alzheimer’s Disease. J. Med. Chem.2022, 65, 4909–492510.1021/acs.jmedchem.1c0215035271276 PMC8958510.^1^*From the template of a multisite AChE inhibitor, we developed a first-in-class multitarget agent with beneficial in vivo effects on neuroinflammation and cognition*.
- GaldeanoC.; ViaynaE.; SolaI.; FormosaX.; CampsP.; BadiaA.; ClosM. V.; RelatJ.; RatiaM.; BartoliniM.; ManciniF.; AndrisanoV.; SalmonaM.; MinguillónC.; González-MuñozG. C.; Rodríguez-FrancoM. I.; Bidon-ChanalA.; LuqueF. J.; Muñoz-TorreroD.Huprine-Tacrine Heterodimers as Anti-Amyloidogenic Compounds of Potential Interest against Alzheimer’s and Prion Diseases. J. Med. Chem.2012, 55, 661–66910.1021/jm200840c22185619 .^2^*In this work, we describe a class of multisite AChE inhibitors, designed inside out, from the CAS to the PAS, and show that they can modulate β-amyloid and prion protein aggregation, beyond AChE*.
- CampsP.; FormosaX.; GaldeanoC.; Muñoz-TorreroD.; RamírezL.; GómezE.; IsambertN.; LavillaR.; BadiaA.; ClosM. V.; BartoliniM.; ManciniF.; AndrisanoV.; ArceM. P.; Rodríguez-FrancoM. I.; HuertasÓ.; DafniT.; LuqueF. J.Pyrano[3,2-*c*]quinoline-6-Chlorotacrine Hybrids as a Novel Family of Acetylcholinesterase- and β-Amyloid-Directed Anti-Alzheimer Compounds. J. Med. Chem.2009, 52, 5365–537910.1021/jm900859q19663388 .^3^*In this work, we describe a class of multisite AChE inhibitors, designed outside in, from the PAS to the CAS, which was later optimized to get a single-digit picomolar inhibitor*.
- CampsP.; El AchabR.; MorralJ.; Muñoz-TorreroD.; BadiaA.; BañosJ. E.; VivasN. M.; BarrilX.; OrozcoM.; LuqueF. J.New Tacrine-Huperzine A Hybrids (Huprines): Highly Potent Tight-Binding Acetylcholinesterase Inhibitors of Interest for the Treatment of Alzheimer’s Disease. J. Med. Chem.2000, 43, 4657–466610.1021/jm000980y11101357 .^4^*This is a pioneering work on the use of molecular hybridization toward an extended binding within the CAS of AChE, which resulted in a class of reversible inhibitors with one of the highest affinities reported at that moment*.

## 1 Introduction

The enzyme acetylcholinesterase (AChE, EC 3.1.1.7) is responsible for the breakdown of neurotransmitter acetylcholine (ACh) into choline and acetate, thereby terminating neurotransmission. Upon hydrolysis of ACh, choline is expelled, and the enzyme becomes acetylated at the catalytic serine. Subsequent hydrolysis of acetylated AChE by a water molecule regenerates the enzyme with the concomitant release of acetic acid. Remarkably, this process takes place at a rate near the diffusion limit, with a turnover of 10^3^–10^4^ s^–1^, which is much higher than that of other serine hydrolases. Indeed, AChE is considered one of nature’s most efficient enzymes.

The extremely high efficiency of AChE and the presence of a permanent positive charge in the trimethylammonium group of ACh anticipated the occurrence of a readily accessible active site, endowed with negatively charged residues in a putative catalytic anionic site (CAS),^5^ but nothing is further from reality. An absolutely unanticipated architecture of the active site was disclosed when the 3D structure of AChE from the Pacific electric ray *Torpedo californica* (TcAChE, PDB entry 2ACE) was solved by Sussman and Silman in 1991.^6^

**Figure 1:**
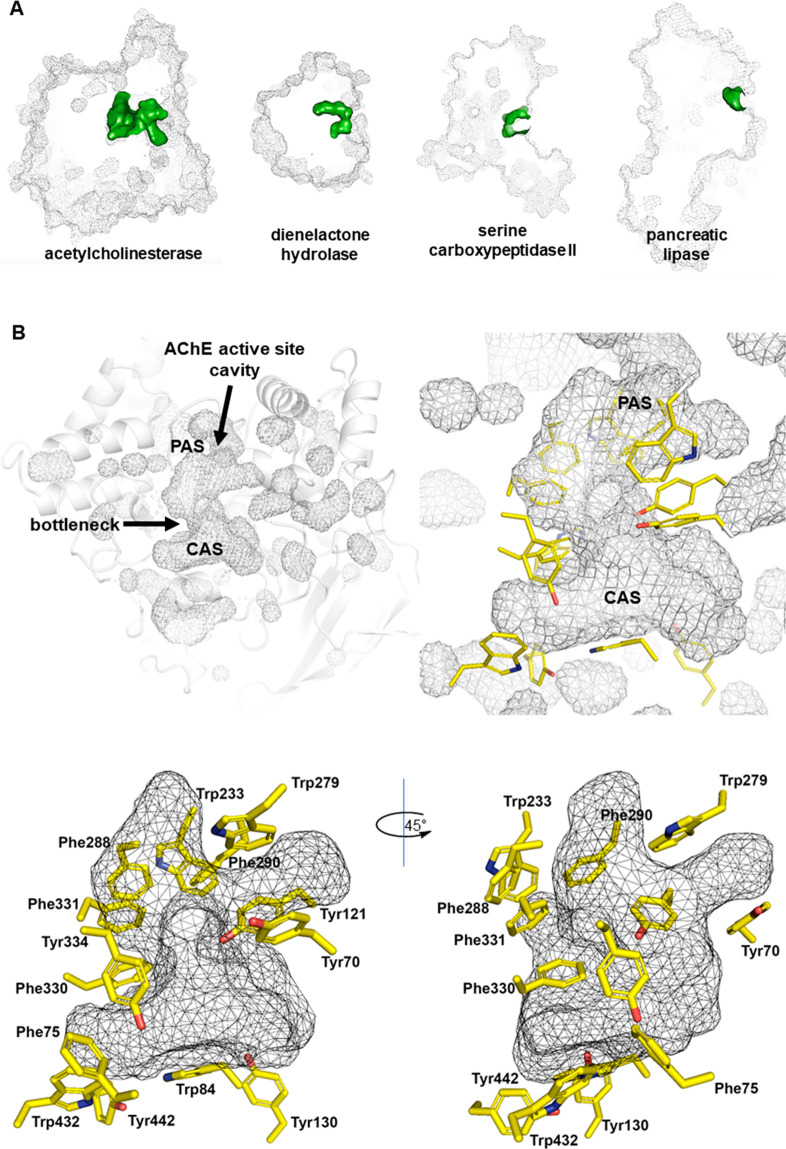
(A) Comparison in size of the active site cavities (in green) of the serine hydrolases *Torpedo californica* acetylcholinesterase (PDB ID 2CKM), *Pseudomonas putida* dienelactone hydrolase (PDB ID 4P92), *Triticum aestivum* serine carboxypeptidase II (PDB ID 3SC2), and *Homo sapiens* pancreatic lipase (PDB ID 1LPB). (B) Representation of the inner cavities in TcAChE and details of the gorge, which is mainly shaped by 14 aromatic residues (sticks, with carbon atoms in yellow).

The active site of this ellipsoidal protein was apparently not so readily accessible. Unlike most enzymes that have active sites located near the surface, the active site of AChE was at the base of a 20-Å-deep gorge that penetrates halfway into the protein (Figure 1A).^6^ At its narrowest point, the gorge has a radius of 5 Å, which is smaller than the diameter of the quaternary ammonium group of ACh (6.4 Å) that must enter through it. After the bottleneck, the gorge widens out at the base (Figure 1B).

AChE displays a negative electrostatic potential that extends over most of the surface, but even if it may be involved in electrostatic attraction and the steering of positive charges into the active site, it does not seem to contribute significantly to the catalytic activity.^7,8^ At the base of the gorge, the “anionic” subsite was indeed neutral and lipophilic,^6^ and the residue interacting with the positive charge of ACh was not an Asp or Glu carboxylate but the indole ring of a Trp residue, namely, Trp84 (TcAChE numbering unless otherwise stated). At the bottleneck, another aromatic residue, Phe330, also contributes to the CAS. Indeed, the presence of aromatic residues is a prominent feature all along the gorge of AChE, which is lined in 70% of its surface by 14 highly conserved aromatic residues. At the mouth of the gorge, Tyr70, Tyr121, and especially Trp279 contribute to the so-called peripheral anionic site (PAS), responsible for the initial transient binding to ACh en route to the CAS.

The access to the deeply confined active site through such a long and abrupt cavity, which might hinder the traffic of the substrate, made counterintuitive the extremely high efficiency of AChE. However, it is precisely the peculiar structure of AChE which accounts for such a high efficiency: (i) the deep confinement of the active site is not an issue; with the active site deeply buried, ACh is almost completely surrounded by the protein, which through an enveloping effect enables multiple substrate–enzyme interactions and hence more effective catalysis; (ii) the long way to the active site and the lack of a negatively charged residue at the “anionic”subsite are not an issue; Asp72 at the rim of the gorge contributes to trapping ACh,^8^ and the high aromatic content of the gorge, from the PAS to the CAS, provides the substrate ACh with aromatic guidance through a low-affinity pathway down to the CAS via successive cation−π interactions; and (iii) the bottleneck before the CAS in not an issue; substantial conformational motions of gorge residues take place to overcome the Phe330/Tyr121 bottleneck, enabling fluent traffic.^9,10^

Not only is AChE a prodigious highly specialized machinery from a physiological point of view but it also offers a vast and challenging territory to be colonized through the design of drugs for diseases that course with a central or peripheral cholinergic deficit, such as Alzheimer’s disease (AD), myasthenia gravis, and glaucoma, among others.

In this Account, we report our efforts to develop anti-AD high-affinity AChE inhibitors, rationally designed to increasingly gain ground in the large active site gorge. Methodologically, this has been undertaken by growing initial hit or lead molecules by molecular hybridization and/or by generating structural complexity by multicomponent reactions. From a design viewpoint, we have addressed the colonization of AChE by gaining extended binding at the CAS first and then designing multisite inhibitors that span the whole length of the gorge from the CAS to the PAS and the other way around, optimizing a PAS binder and then colonizing the gorge from the PAS to the CAS. We also discuss how the molecular template provided by AChE enables the design of compounds that fit well within other key proteins in AD, such as BACE-1 and amyloidogenic proteins, and how it can be used to rationally design AChE inhibitor-based multitarget agents that modulate key pathogenic events of AD, such as oxidative stress and neuroinflammation.

## 2 Colonization of the Active Site

**Figure 2:**
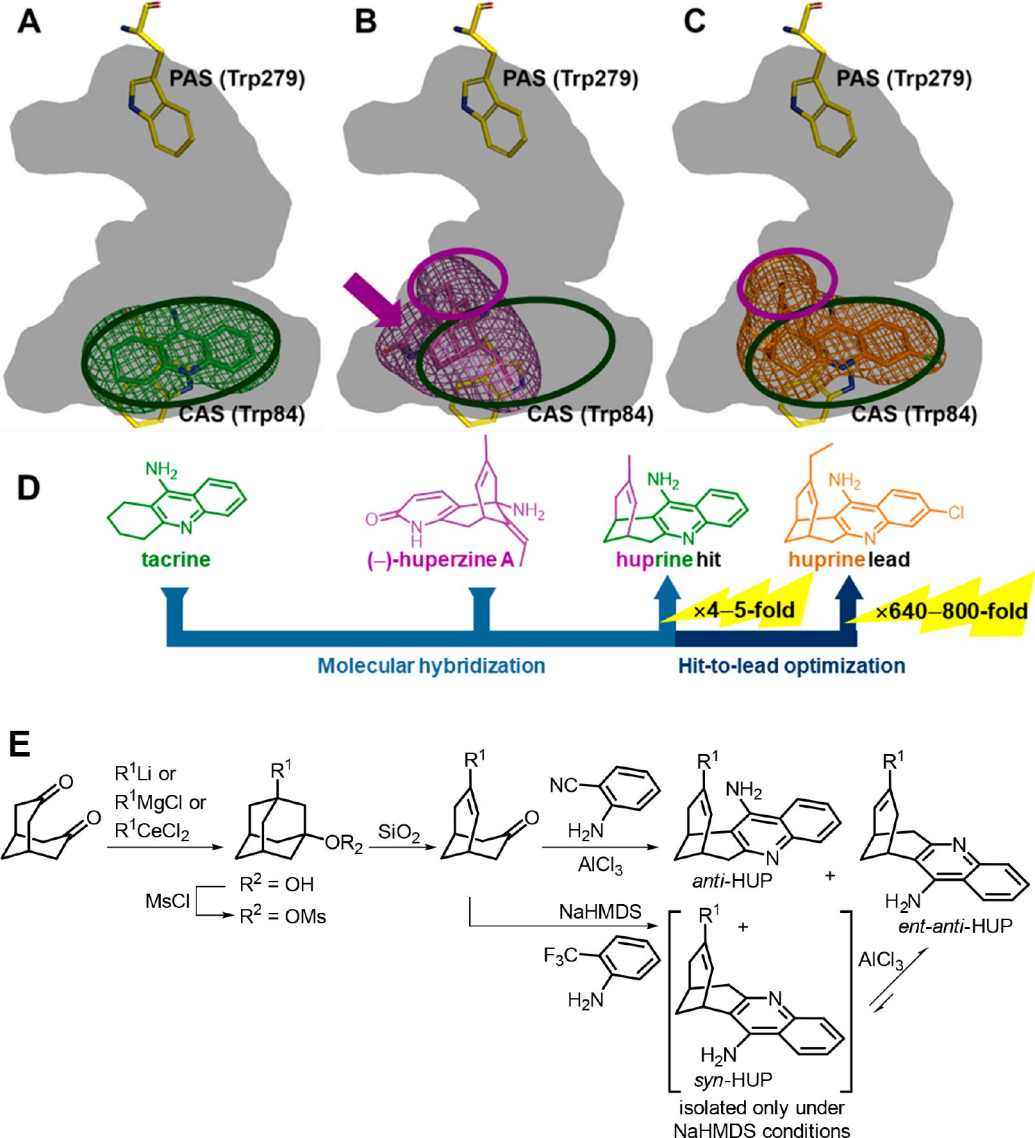
Placement of tacrine (A), (−)-huperzine A (B), and (−)-huprine X (C) at the CAS of TcAChE (PDB ID 2ACJ, 1VOT, and 1E66, respectively): inner surface of the active site gorge in dark gray and inhibitors in sticks with surrounding occupied volume in mesh (the same applies for similar views in the following figures). For comparison purposes, dark-green and purple ellipses show the regions occupied by tacrine and the unsaturated bridge of (−)-huperzine A, respectively, and the purple arrow shows the region occupied by the pyridone ring of (−)-huperzine A. (D) Design of huprines, with an indication of the potency increase relative to tacrine and (−)-huperzine A. (E) Synthesis of huprines.^4,13,14^

In 1993, Sussman and Silman solved the crystal structure of TcAChE in complex with tacrine, the first approved anti-AD drug,^11^ and 4 years later the structure of the complex with the natural product (−)-huperzine A.^12^ Tacrine and (−)-huperzine A occupy adjacent binding sites within the CAS of AChE, which overlap only partially (Figure 2A,B). Thus, the cyclohexene moiety of (−)-huperzine A fused to the pyridone ring is accommodated in the same site as the cyclohexene ring of tacrine, whereas the methyl-substituted unsaturated three-carbon bridge and the pyridone ring of (−)-huperzine A fill other regions within the CAS that are not occupied by tacrine.

This left room for designing molecules to occupy the common and noncommon binding sites of tacrine and (−)-huperzine A, increasing the number of contacts with AChE and thereby the inhibitory potency. Our group pioneered the use of molecular hybridization to develop high-affinity inhibitors with extended binding at the CAS. Integration of the methyl-substituted unsaturated three-carbon bridge of (−)-huperzine A onto the cyclohexene ring of tacrine led to a class of huperzine A–tacrine hybrids (Figure 2D). With an IC_50_ of 52 nM, the initial hit was a 4- and 5-fold more potent inhibitor of human AChE (hAChE) than tacrine and (−)-huperzine A, respectively.^4,13^ After hit-to-lead optimization, the levorotatory (7*S*,11*S*)-enantiomers of the 3-chloro-9-methyl and 3-chloro-9-ethyl derivatives (huprines Y and X) exhibited subnanomolar potencies (IC_50_ = 0.32 nM) in hAChE, being 640- and 800-fold more potent than tacrine and (−)-huperzine A, respectively.^4^ Terrone L. Rosenberry, who coined the term huprines as a more practical nickname than **hup**erzine–tac**rine** hybrids, found that with inhibition constants (*K*_I_) of 26 and 33 pM, huprine X and huprine Y were among the highest-affinity reversible inhibitors reported for hAChE.^15^ The 3D structure of the complex TcAChE–huprine X confirmed the wider occupancy of the CAS by huprines relative to the parent compounds (Figure 2C) with (i) the aminoquinoline system in the same binding site as tacrine, stacking between Trp84 and Phe330 and establishing H-bonds with His440 and a network of water-mediated contacts through its protonated pyridine nitrogen and the primary amino group, respectively; (ii) the carbobicyclic moiety occupying a very similar volume of the CAS as the corresponding fragment in (−)-huperzine A; and (iii) the chlorine atom further extending the binding at CAS by filling a hydrophobic pocket.^16^

**Figure 3:**
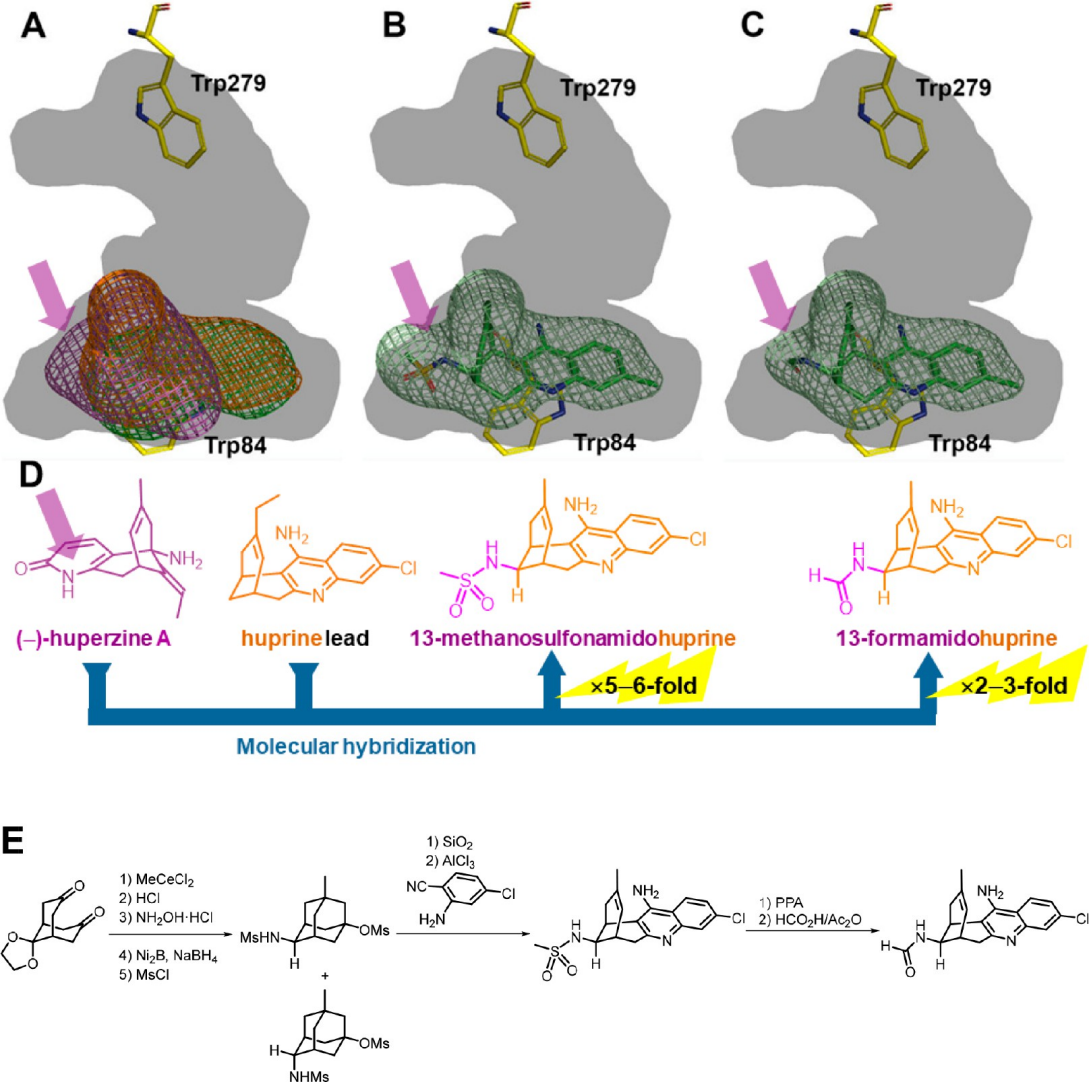
Superposition of the volumes occupied by tacrine, (−)-huperzine A, and huprine X within the gorge of TcAChE (A) and placement of 13-methanosulfonamidohuprine (B) and 13-formamidohuprine (C), based on MD simulations. The purple arrow shows the region occupied by the pyridone ring of (−)-huperzine A and its surrogate moieties in 13-amidohuprines. (D) Design of 13-amidohuprines, with an indication of the potency increase relative to tacrine and (−)-huperzine A. (E) Synthesis of 13-amidohuprines.^18^

**Figure 4:**
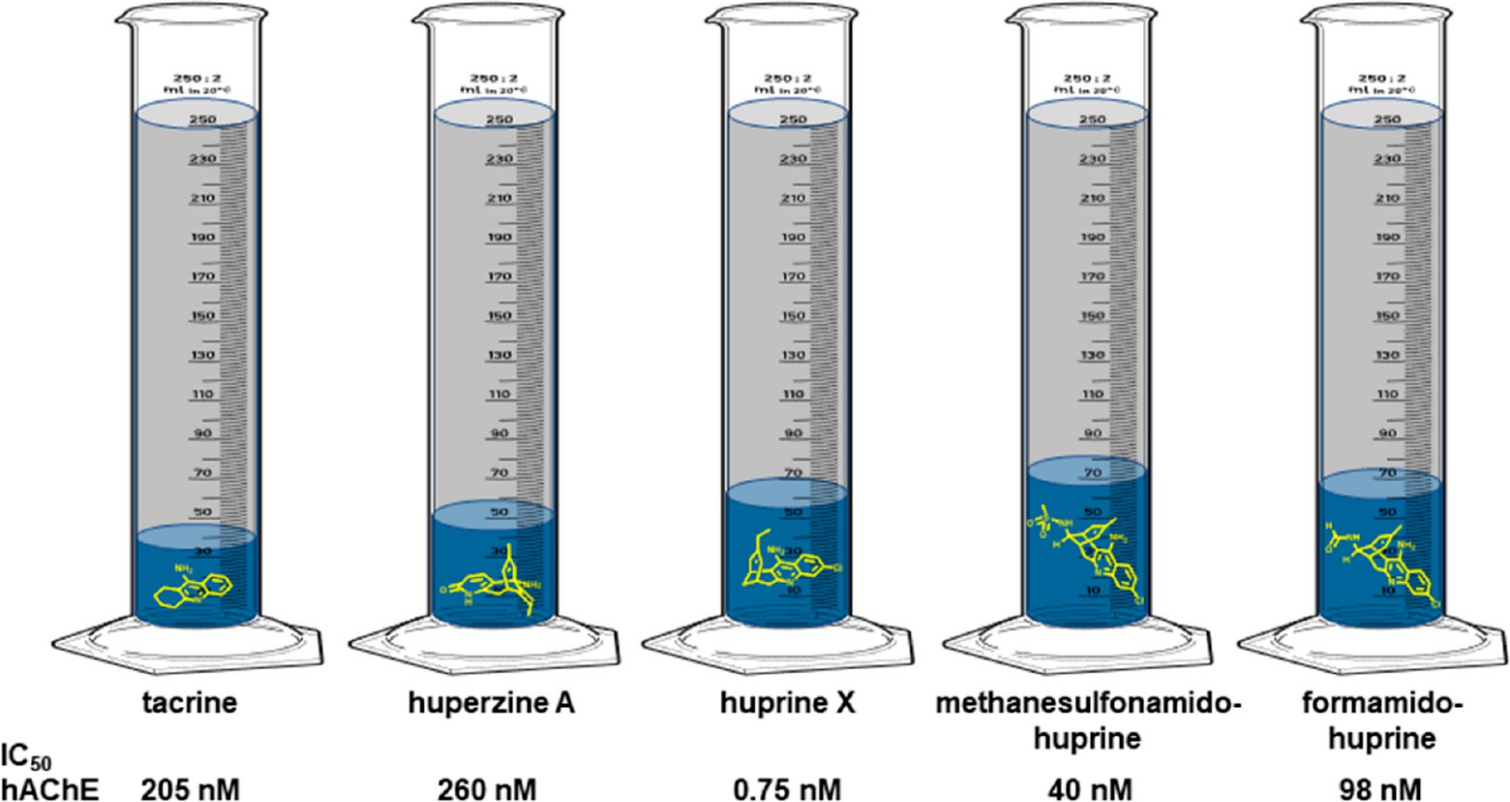
Illustration of the volume of the hAChE gorge (in gray + blue) occupied by CAS inhibitors designed by molecular hybridization from tacrine and huperzine A (volumes in blue, chemical structures in yellow).

As a second step to further extend binding at the CAS, we explored growing huprines to reach the binding region of the pyridone ring of (−)-huperzine A (purple arrow in Figure 2B and Figure 3A). When (−)-huperzine A binds to AChE, the Gly117–Gly118 peptide bond suffers a conformational flip to avoid a steric clash between the pyridone carbonyl and the backbone carbonyl of Gly117. To gain full occupancy of the binding sites of tacrine and (−)-huperzine A at the CAS, we introduced different amido groups at the methylene bridge (position 13) of huprine Y with the two possible diastereomeric arrangements, which were to mimic the pyridone C(O)NH group of (−)-huperzine A.^17,18^ Molecular dynamics (MD) simulations suggested that both 13-formamido and 13-methanesulfonamido-huprines could trigger the Gly117–Gly118 conformational flip, filling a larger volume of the CAS than that of huprine Y (Figure 3B,C). However, despite being 2–5- and 3–6-fold more potent hAChE inhibitors than tacrine and (−)-huperzine A, respectively, 13-formamido and 13-methanesulfonamidohuprines were less potent than huprine Y (Figure 4). The gain in interaction energy due to the additional contacts formed by the amido groups seemed to be overcome by the deformation cost associated with the Gly117–Gly118 flip, making the net balance of the binding affinity of 13-amidohuprines unfavorable relative to that of huprine Y.

## 3 Colonization of the Active Site Gorge

### 3.1 From the CAS to the PAS of AChE

At the time we were developing huprines, Pang et al. identified by computational studies the PAS of AChE as a low-affinity binding site of tacrine and developed a series of alkylene-linked tacrine dimers, designed to simultaneously block the CAS and the PAS, by placing one tacrine unit at each binding site, reaching potency increases of up to 57-fold relative to tacrine.^19,20^

**Figure 5:**
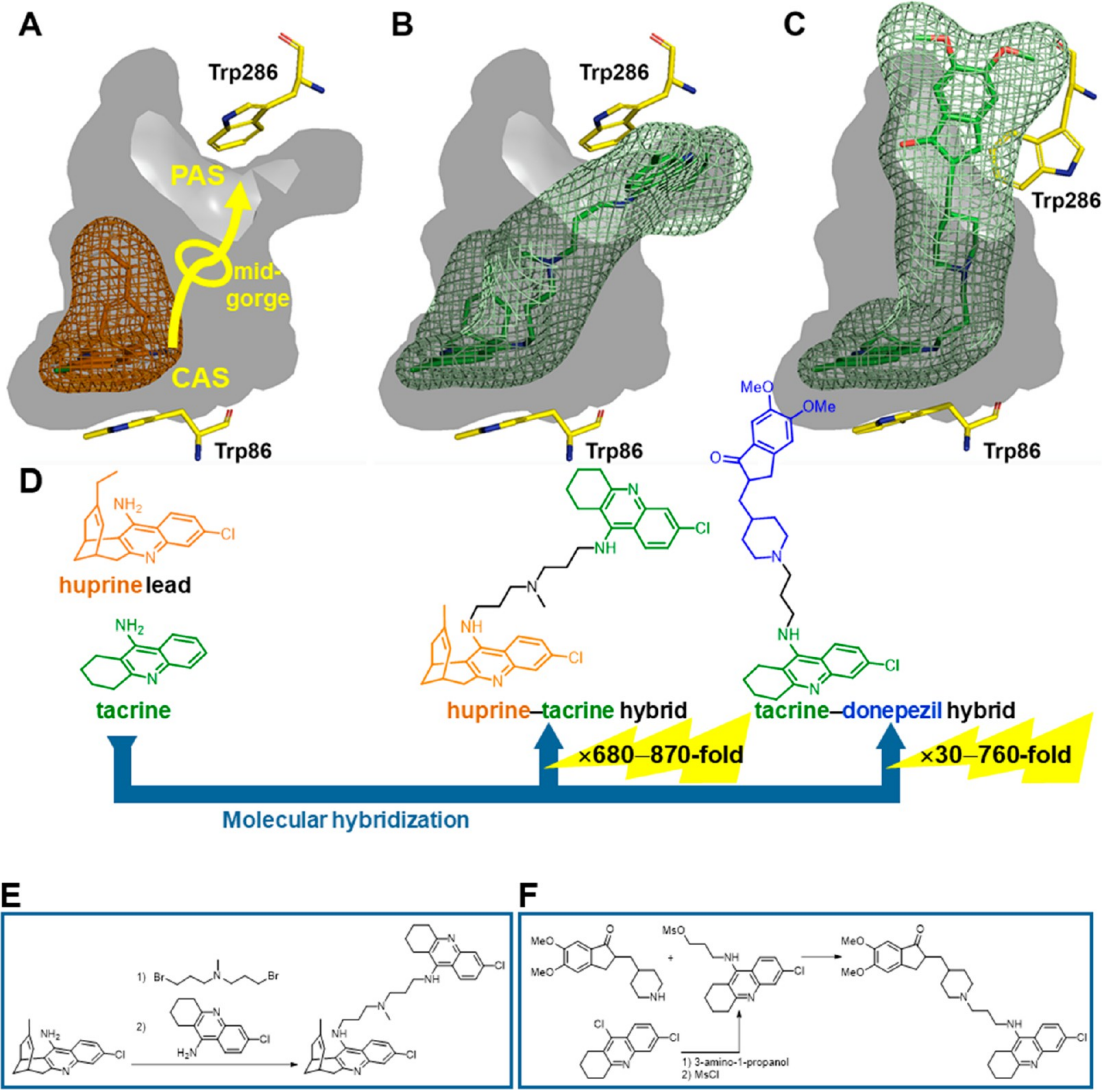
(A) Design strategy to grow up huprine or tacrine to span the whole length of the AChE gorge, from the CAS to the PAS, while gaining interactions at midgorge (hAChE numbering). Placement of the lead huprine–tacrine (B) and tacrine–donepezil (C) hybrids within the gorge, based on MD simulations. (D) Design of these multisite AChE inhibitors, with an indication of the potency increase relative to tacrine and (−)-huperzine A and synthesis thereof (E, F).^2,22^

**Figure 6:**
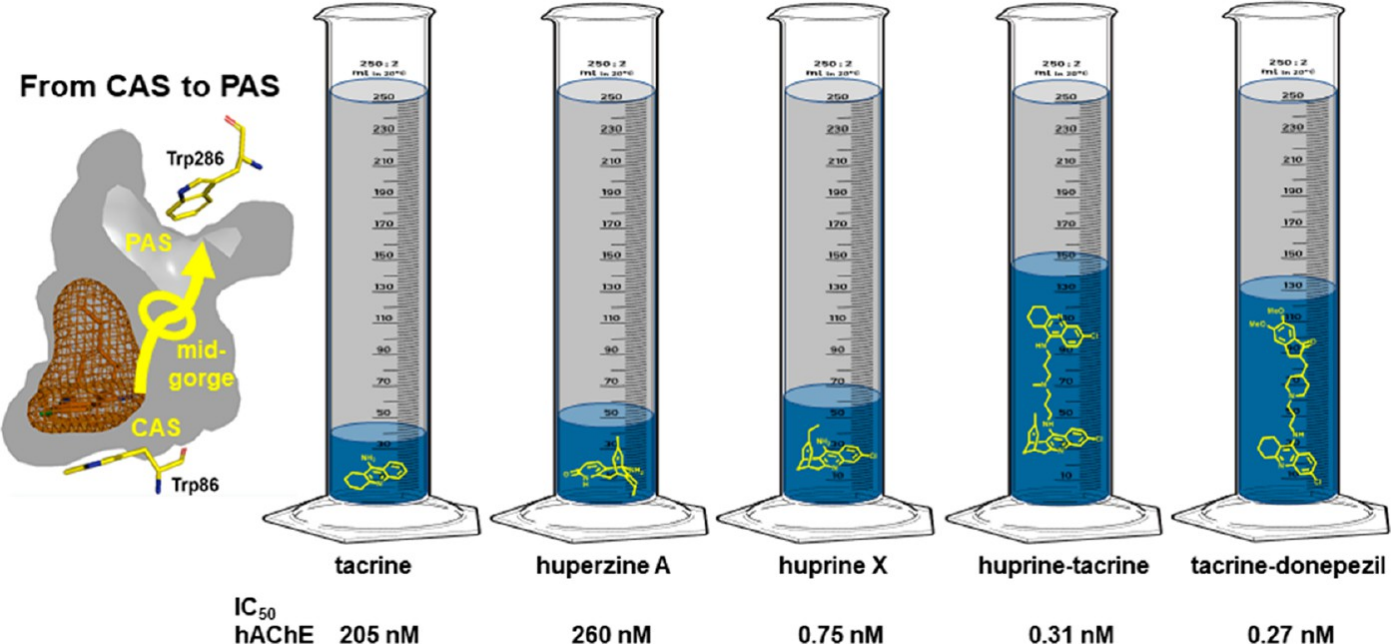
Illustration of the volume of the hAChE gorge (in gray + blue) occupied by huprine- and tacrine-based multisite inhibitors compared with the parent compounds (volumes in blue, chemical structures in yellow).

In light of these results, at the next step in our journey toward high-affinity AChE inhibitors by increasing the occupancy of the AChE gorge through molecular hybridization, we grew up huprines by linking a tacrine unit, as a PAS-binding moiety, through suitable tether chains, guided by MD simulations (Figure 5A). Apart from bridging the distance from the CAS to the PAS, the linker was used to establish additional interactions. Indeed, the introduction of a protonatable amine within the linker enabled cation−π interactions with midgorge aromatic residues,^21^ as a third binding site, in addition to CAS and PAS. The resulting multisite ligands exhibited subnanomolar potencies (IC_50_ around 0.3 nM, in racemic form), which represented an increase in hAChE inhibitory potency of 680- and 870-fold relative to tacrine and (−)-huperzine A and a meritorious 3-fold increase relative to huprine Y (Figure 5B,D, Figure 6).^2^

Likewise, hybridization of the CAS inhibitor 6-chlorotacrine with the 5,6-dimethoxy-2-[(4-piperidinyl)methyl]-1-indanone moiety of the anti-AD drug donepezil resulted in subnanomolar multisite inhibitors, whose protonated piperidine nitrogen and indanone system served as the midgorge and PAS-anchorage points, through a salt bridge with Asp74 and π-stacking with Trp286 (hAChE numbering), respectively.^22^ Considering the IC_50_ of 0.27 nM of the best 6-chlorotacrine–donepezil hybrid, this hybridization resulted in gains of potency of 760-, 30-, and 45-fold over tacrine, 6-chlorotacrine, and donepezil, respectively (Figure 5C,D, Figure 6).

### 3.2 From the PAS to the CAS of AChE

Multisite AChE inhibitors are usually built starting from a potent CAS inhibitor and growing the molecule to reach the PAS with a structural motif that could be engaged in π-stacking or cation−π interactions with Trp279 (Trp286 in hAChE). Highly potent multisite AChE inhibitors can also be built the other way around, by growing a PAS ligand to gain gorge occupancy from the PAS to the CAS. Using a Povarov multicomponent reaction,^23^ we synthesized a pyrano[3,2-*c*]quinoline derivative, structurally close to the PAS inhibitor propidium, which displayed low hAChE inhibitory potency (IC_50_ > 10 μM),^3^ likely arising from π-stacking interactions with Trp286. A bioisosteric replacement of the oxygen atom of the pyrano[3,2-*c*]quinoline scaffold of this hit by a NH group increased the basicity of the quinoline nitrogen of the resulting tetrahydrobenzo[*h*][1,6]naphthyridine, which should be protonated at physiological pH, thereby enabling cation−π interactions in addition to π-stacking, reinforcing the interaction with the PAS Trp286. A second O → NH bioisosteric replacement at the side chain led to an amido group, which snaked down the initial portion of the gorge, enabling H-bond interactions with midgorge residues (Tyr124, hAChE numbering). The optimized benzonaphthyridine PAS binder displayed a remarkable potency in hAChE (IC_50_ = 65 nM).^24^

**Figure 7:**
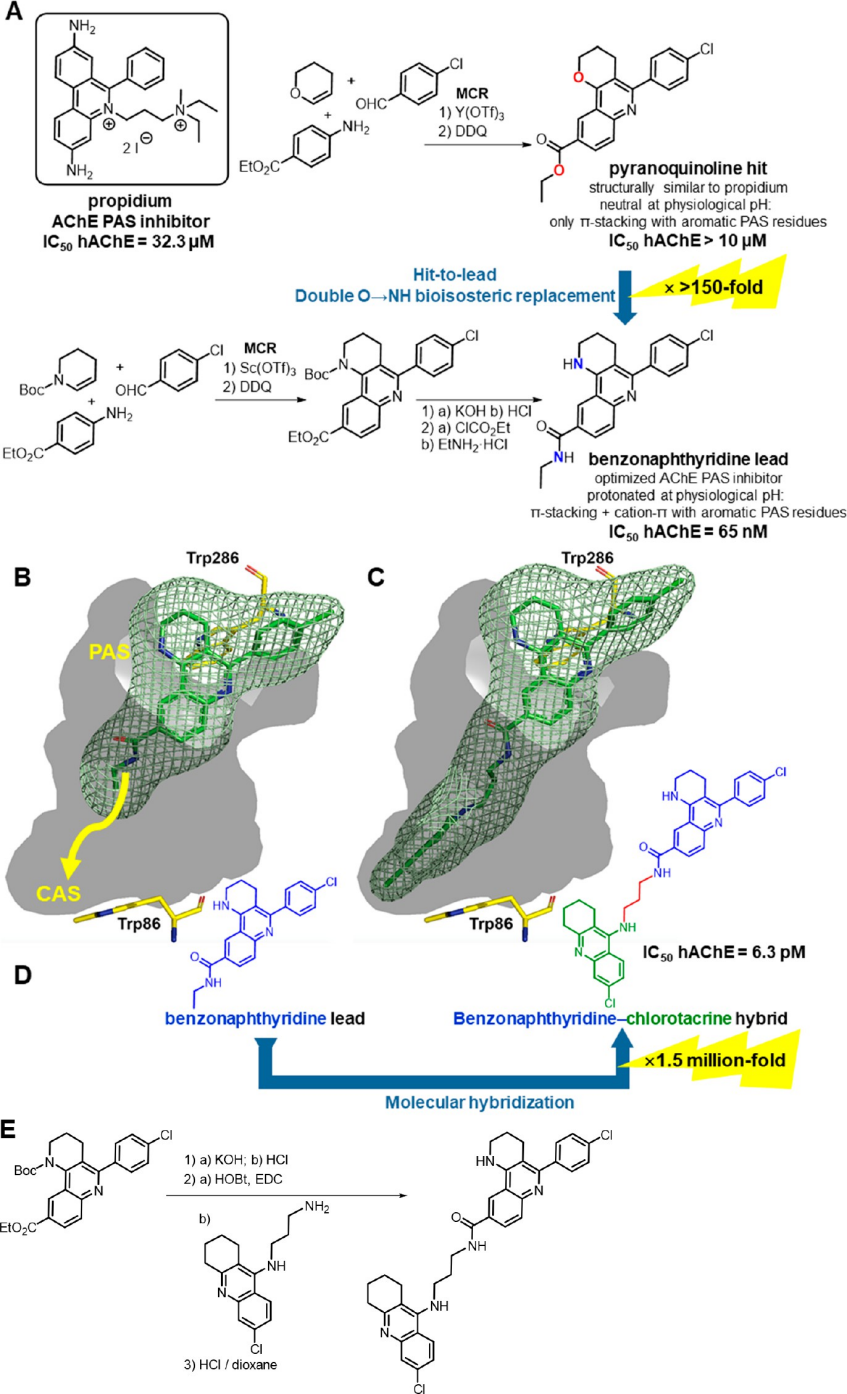
(A) Design and synthesis of PAS binders. (B) Design strategy to grow the benzonaphthyridine lead to span the whole length of the AChE gorge, from the PAS to the CAS (hAChE numbering). (C) Placement of the benzonaphthyridine–chlorotacrine hybrid within the gorge, based on MD simulations, and its design (D), with an indication of the potency increase relative to the initial pyranoquinoline hit. (E) Synthesis of the multisite inhibitor.^25^

**Figure 8:**
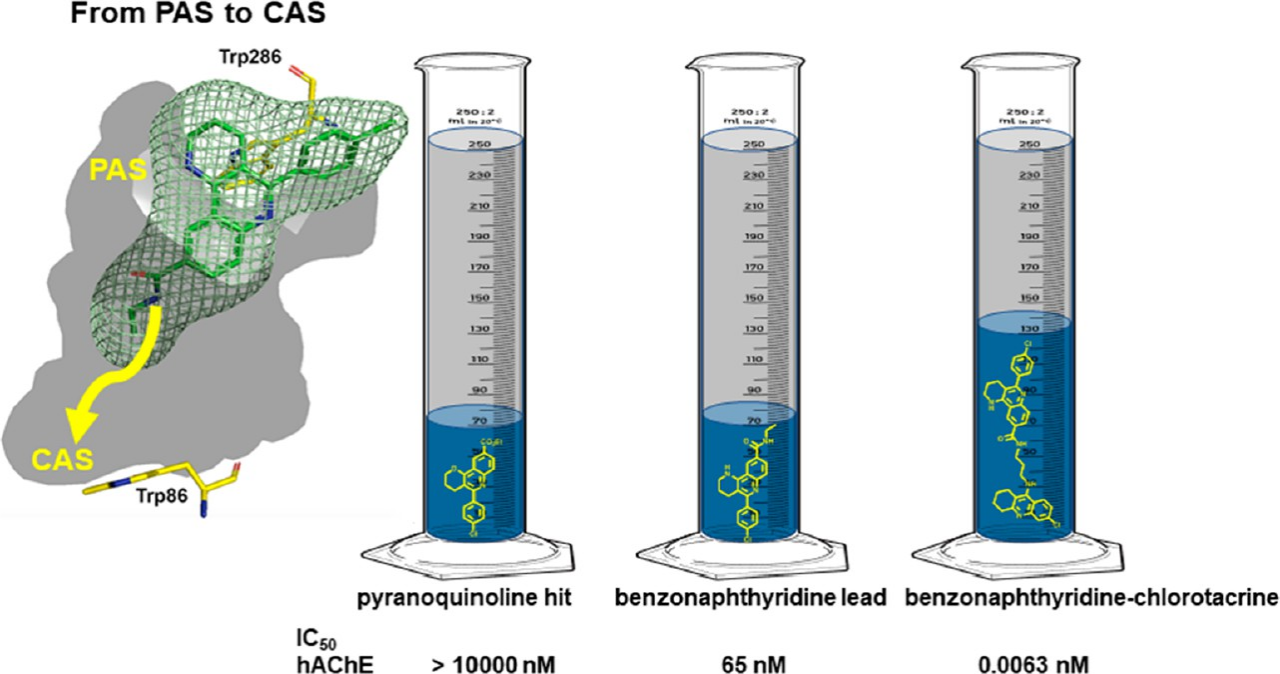
Illustration of the volume of the hAChE gorge (in gray + blue) occupied by the benzonaphthyridine–chlorotacrine multisite inhibitor compared with the PAS binders (volumes in blue, chemical structures in yellow).

Because the side-chain amido group points downward in the AChE gorge, we grew the PAS binder lead to extend the gorge occupancy from the PAS to the CAS by linking it to a potent CAS binder, such as 6-chlorotacrine (Figure 7B,C). Guided by MD simulations, a trimethylene linker afforded the optimal distance to span the last portion of the gorge and place the 6-chlorotacrine unit stacked against the CAS Trp86 (hAChE numbering). Indeed, the resulting hybrid featured an impressive IC_50_ of 6 pM in hAChE, which represents a gain of potency >1.5 million-fold relative to the initial pyranoquinoline hit, >10.000-fold relative to the benzonaphthyridine lead, and 1000-fold relative to 6-chlorotacrine (Figure 8).^25^ This example illustrates how powerful molecular hybridization can be to gain occupancy of the AChE gorge and maximize the number of drug–target contacts.

Sharpless and collaborators developed a very elegant alternative manner to build AChE multisite inhibitors of extremely high affinity from a CAS and a PAS binder, decorated with alkylene chains containing bioorthogonal complementary reactive groups, which, upon incubation with AChE, react to covalently assemble both moieties, directly within the enzyme, which is used as the reaction vessel and catalyst (*in situ* click chemistry). In the original work, tacrine and propidium-like phenylphenanthridinium moieties were used as the CAS and PAS binders, respectively. They contained tether chains of variable length terminated in an azide in one case and in an alkyne in the other to enable a Huisgen 1,3-dipolar cycloaddition reaction that connects the CAS and the PAS binding moieties through the formation of a triazole ring, which finally appears within the linker of the assembled multisite inhibitor. This reaction is extremely slow at room temperature, in the absence of catalyst, due to a very high activation barrier (ca. 25 kcal/mol). The incubation of binary or more complex mixtures of azides and acetylenic reagents with electric eel or mouse AChE at room temperature for 6 days led to the recruitment of some specific CAS/PAS binder pairs, whose azide and alkyne groups were placed in close proximity in a particular environment within the active site gorge, that (i) stabilized the triazole-like transition state, thereby causing a tremendous rate acceleration and (ii) enabled additional midgorge interactions with the formed triazole ring of the hybrids, apart from those at CAS and PAS, thereby contributing to the amazing femtomolar potencies of the *in situ*-assembled multisite inhibitors.^26,27^

## 4 Beyond AChE: Templating Modulators of Other Proteins

The structural requirements for multisite AChE inhibitors imposed by the AChE gorge shape a particular type of molecule that can inherently modulate other key targets in AD, such as BACE-1 and β-amyloid (Aβ) and tau aggregation. Moreover, one of the structural moieties of multisite AChE inhibitors can be chosen to satisfy both the stereoelectronic requirements for interactions with one of the binding sites of AChE (usually the PAS) and the pharmacophoric elements for interaction with another target, leading to rationally designed multitarget compounds.

### 4.1 Multisite AChE Inhibitors That Modulate BACE-1

BACE-1 is an aspartate protease that mediates the first and most rate-limiting step of the formation of Aβ from the amyloid precursor protein (APP). Because Aβ is one of the main culprits of AD, BACE-1 is a prime target for AD treatment. Unlike the buried AChE catalytic site, the active site of BACE-1 is an open cleft, but both of them have in common that they are long cavities with several binding sites,^28^ thereby enabling multisite binding.

**Figure 9:**
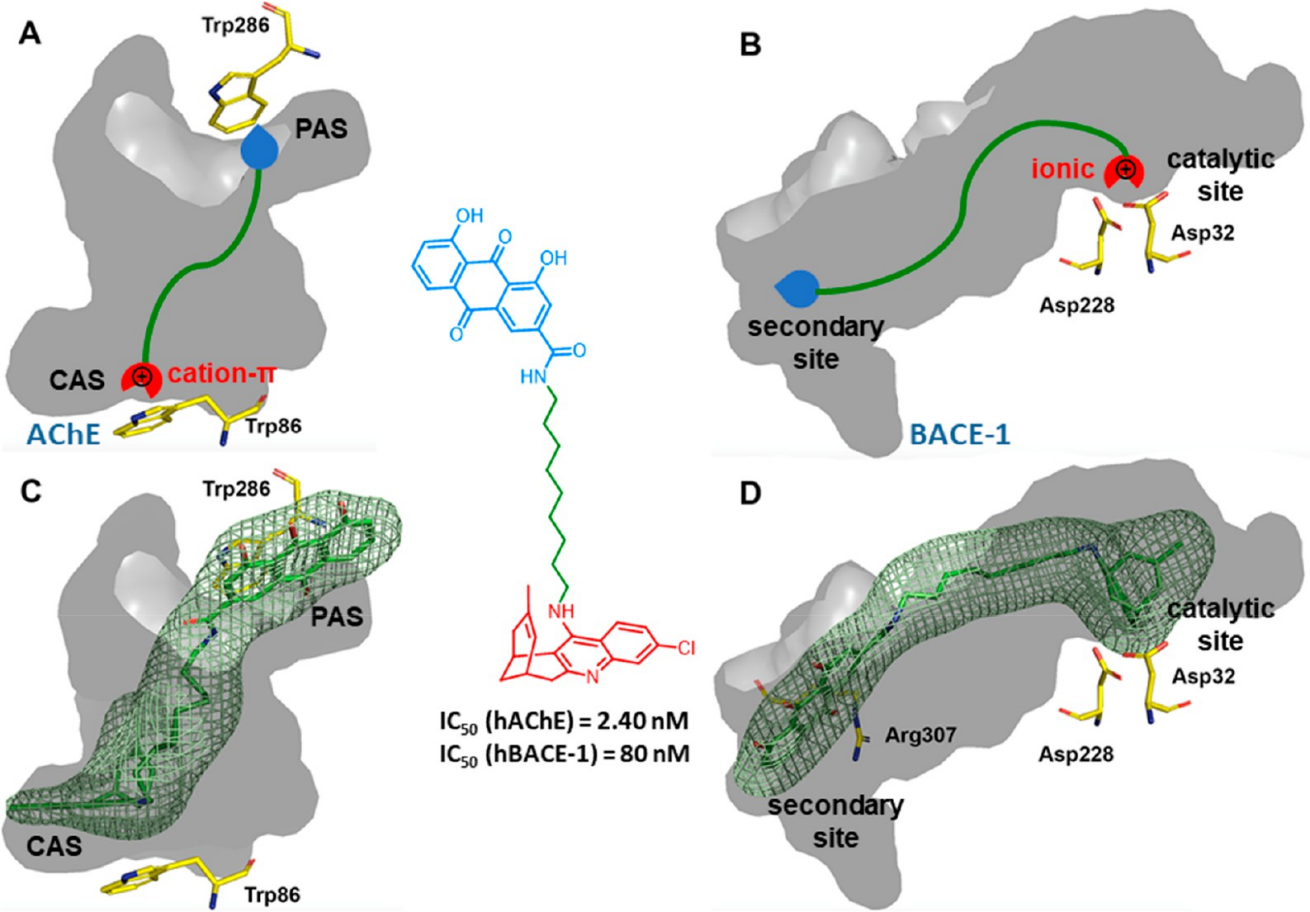
Structural requirements for multisite interaction within AChE (A) and BACE-1 (B) (human enzymes numbering). Placement of the rhein–huprine hybrid within the active site cavities of AChE (C) and BACE-1 (D), based on MD simulations.

2-Aminopyridine and 2-aminoquinoline are known binding motifs that, in charged form, engage the catalytic Asp residues of BACE-1.^29^ The introduction of flexible aliphatic chains and second-site fragments that extend off of the heteroaromatic ring has been used to occupy additional binding sites and increase the inhibitory potency in BACE-1.^30^ This situation might be paralleled in tacrine- and huprine-based multisite AChE inhibitors, in which (i) the protonated 4-aminoquinoline moiety that interacts with the AChE CAS might also engage the Asp dyad of BACE-1 and (ii) the linked moiety that spans the AChE gorge and reaches the PAS might extend beyond the catalytic dyad of BACE-1 to occupy additional pockets, thereby leading to a multisite binding also in BACE-1 (Figure 9A,B). Indeed, several classes of tacrine- and huprine-based hybrids and other structurally related multisite AChE inhibitors have been found to inhibit BACE-1, usually with micromolar to nanomolar potency.^2,3,31,32^ For example, a rhein–huprine hybrid, developed as a multisite AChE inhibitor, inhibits BACE-1 with an IC_50_ of 80 nM,^33^ although neither rhein nor huprine Y has significant activity on this enzyme. The high potency of this hybrid, with more than 100-fold enhanced affinity relative to that of the parent compounds, was indicative of a highly synergistic cooperative effect arising from a precise arrangement of the rhein and huprine moieties of the hybrid in the cleft of BACE-1. MD simulations and pocket druggability studies suggested the transient opening of a novel secondary site at the edge of the catalytic cleft, in which the rhein moiety of the hybrid was placed, H-bonded to Arg307, while the protonated huprine unit was interacting with the Asp dyad.^34^ Thus, this rhein–huprine hybrid behaves as a multisite inhibitor of both AChE and BACE-1 (Figure 9C,D).

### 4.2 Multisite AChE Inhibitors That Modulate Aβ and Tau Aggregation

**Figure 10:**
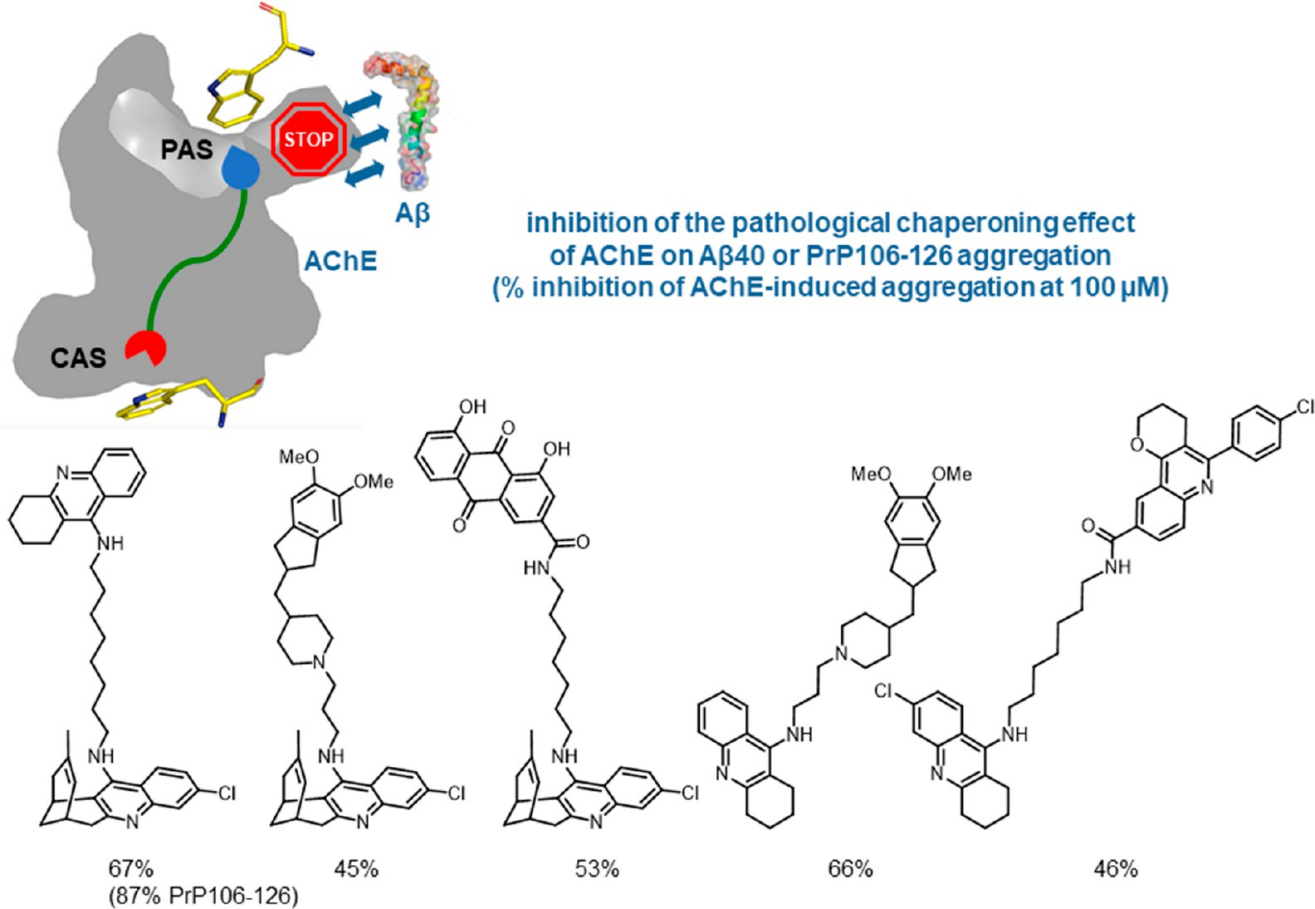
Structures of multisite inhibitors of AChE and their inhibitory activity of AChE-induced Aβ40 or PrP106–126 aggregation.

AChE can interact through the PAS with Aβ, promoting a conformational change that accelerates Aβ aggregation and increases its neurotoxicity.^35^ AChE can have a similar chaperoning effect on prion protein (PrP) aggregation, which might have relevance both in prion diseases and in AD. Thus, AChE promotes the aggregation of PrP106–126,^36^ a key fragment for PrP aggregation, of PrP82–146,^37^ the major component of amyloid plaques in Gerstmann–Sträussler–Scheinker disease, and of full-length PrP,^38^ in the latter case increasing its cytotoxicity in primary neuronal cultures. Blockade of the AChE PAS by multisite inhibitors would prevent these chaperoning effects by occluding the AChE–Aβ and AChE–PrP interfaces. Indeed, multisite AChE inhibitors do inhibit in vitro the AChE-induced aggregation of Aβ40, whereas CAS inhibitors, such as tacrine and 6-chlorotacrine, do not.^2,3,22,39,40^Figure 10 shows some multisite AChE inhibitors developed in our group that inhibit AChE-induced Aβ40 and PrP106–126 aggregation.

Likewise, the huprine–tacrine hybrid inhibited in vitro the AChE-induced aggregation of PrP106–126 by 87% at 100 μM^2^ and of monomeric full-length PrP by 50% at 1.25 μM and dose-dependently reduced the accumulation of the pathological misfolded prion protein conformer PrP^Sc^ in prion-infected MovS6 cells at 0.1 to 0.5 μM concentrations.^38^

**Figure 11:**
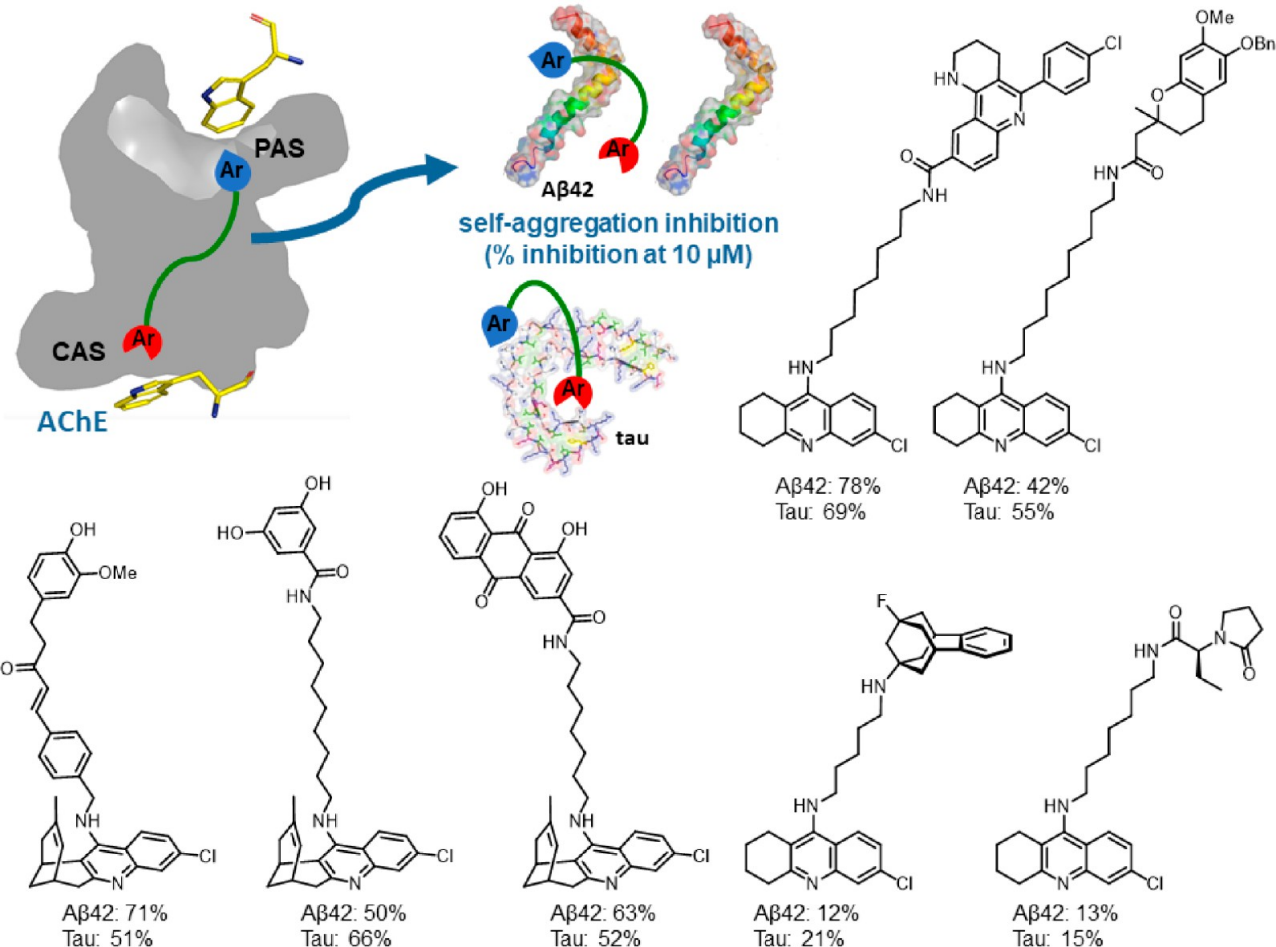
Structures and Aβ42 and tau antiaggregating activity of multisite AChE inhibitors.

Multisite AChE inhibitors can block the AChE-induced Aβ aggregation and also inhibit Aβ self-aggregation. The presence of Trp residues at the CAS and PAS of AChE drives the design of multisite inhibitors toward dimeric or hybrid compounds usually containing (hetero)aromatic rings at both ends of a linker to enable π–π interactions. We have consistently found that these compounds inhibit Aβ42 aggregation in vitro^2,3^ and in *Escherichia coli* cells that overexpress Aβ42 (Figure 11).^41−45^ Most of these compounds inhibit Aβ42 aggregation in the range of 40–80% at 10 μM, whereas multisite AChE inhibitors without an aromatic ring at one of its ends or with an aromatic ring fused to a nonplanar (polycyclic) ring system are essentially inactive.

**Figure 12:**
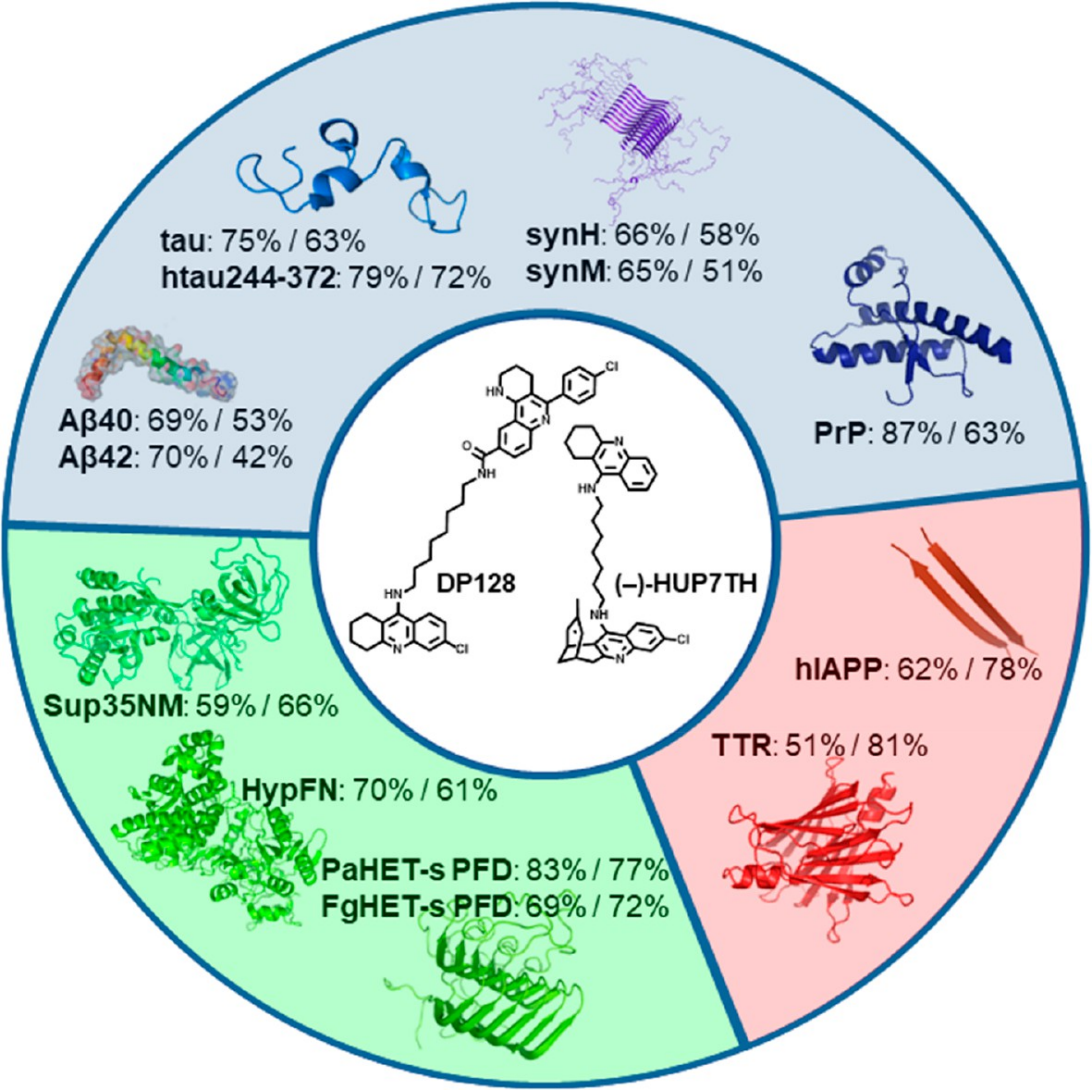
Effect of amyloid pan inhibitors on the aggregation of proteins involved in neurological diseases, such as Aβ40, Aβ42, tau protein, truncated tau protein that retains the amyloid-prone region htau244–372 (AD and other tauopathies), human and mouse α-synuclein (synH, synM) (Parkinson’s disease), and PrP (spongiform encephalopathies), involved in non-neurological diseases such as transthyretin (TTR) (transthyretin-related amyloidosis) and human islet amyloid polypeptide (hIAPP) (type-2 diabetes), and fungal, yeast, and bacterial amyloids, such as the PaHET-s prion-forming domain (PaHET-s PFD), Fg-HET-s PFD, Sup35NM, and HypF-N.

In AD, another amyloidogenic protein apart from Aβ, tau protein, plays a key pathogenic role. This makes the inhibition of Aβ and tau aggregation an attractive mechanism of action for the disease-modifying treatment of AD.^46^ Multisite AChE inhibitors consisting of two (hetero)aromatic rings linked through a suitable tether also inhibit tau aggregation, usually with potencies similar to those found for Aβ42 aggregation (Figure 11). The dual antiaggregating activity might be ascribed to the presence of similar cross-β motifs and the occurrence of similar aggregation mechanisms. Along this line, we inferred that compounds capable of inhibiting the formation of β-sheet structures could abolish the aggregation of other amyloids, behaving as amyloid pan-inhibitors. By using *E. coli* cells genetically modified to separately express 13 amyloid-prone proteins (Figure 12 legend), involved in neurological and non-neurological diseases, as well as fungal, yeast, and bacterial amyloidogenic proteins, we found that the multisite AChE inhibitors DP128 and (−)-HUP7TH block the aggregation of all of the tested amyloidogenic proteins, with similar potencies (50–85% and 40–80%, respectively, at 10 μM).^47^ In contrast, the CAS inhibitor huprine Y was essentially inactive against all amyloids. Thus, these compounds might interact through a general mechanism with different amyloids, opening new avenues for the treatment of amyloidoses.

### 4.3 Multisite AChE Inhibitor-Based Multitarget Compounds

**Figure 13:**
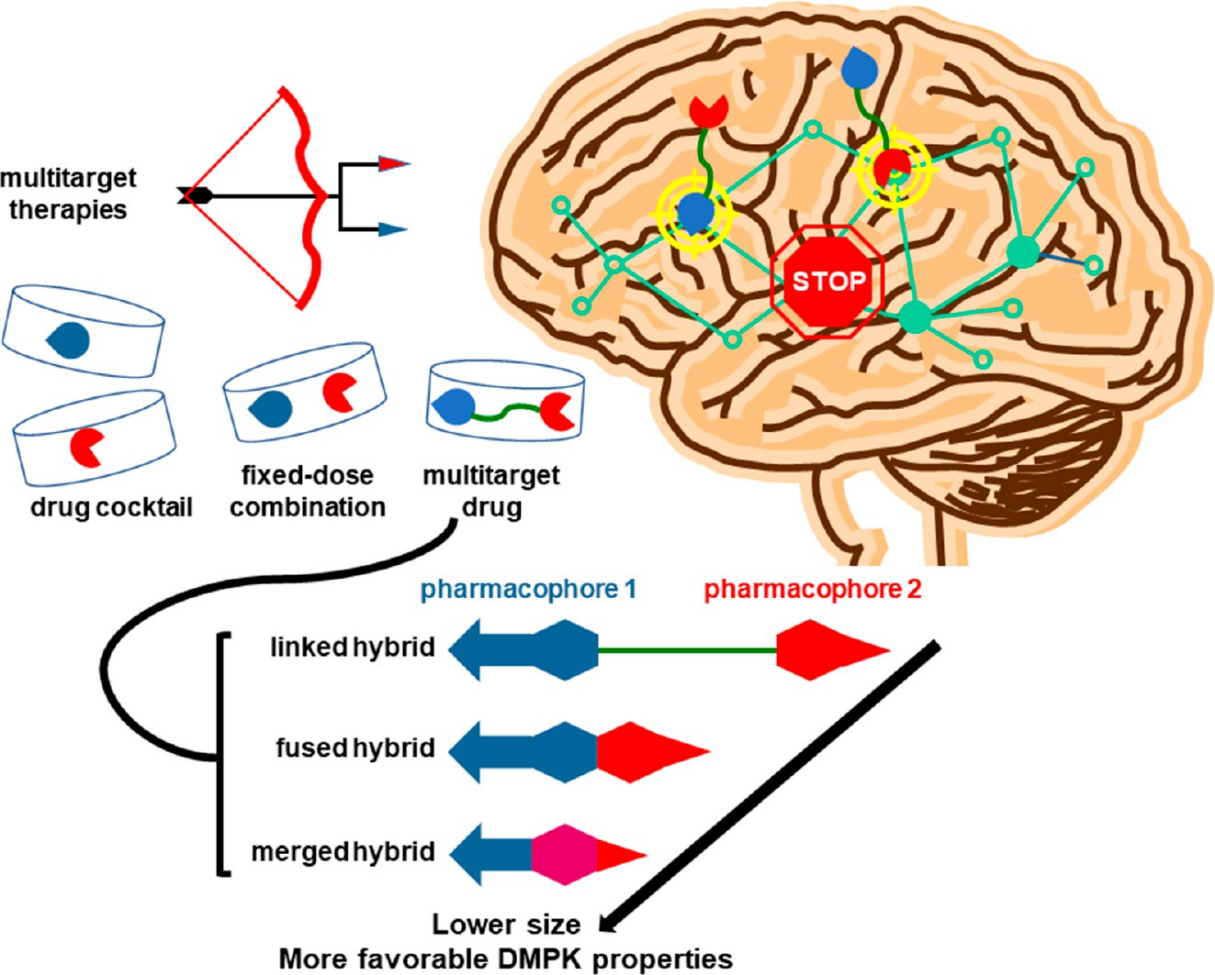
Different modalities of multitarget therapies and design strategies for multitarget drugs.

Multitarget compounds, which combine two pharmacophores, each one intended for the modulation of a different target, should lead to a more efficient management of multifactorial diseases.^48,49^ Both pharmacophores can be fused, merged if they contain common substructures, or connected through a linker (Figure 13).^48^

**Figure 14:**
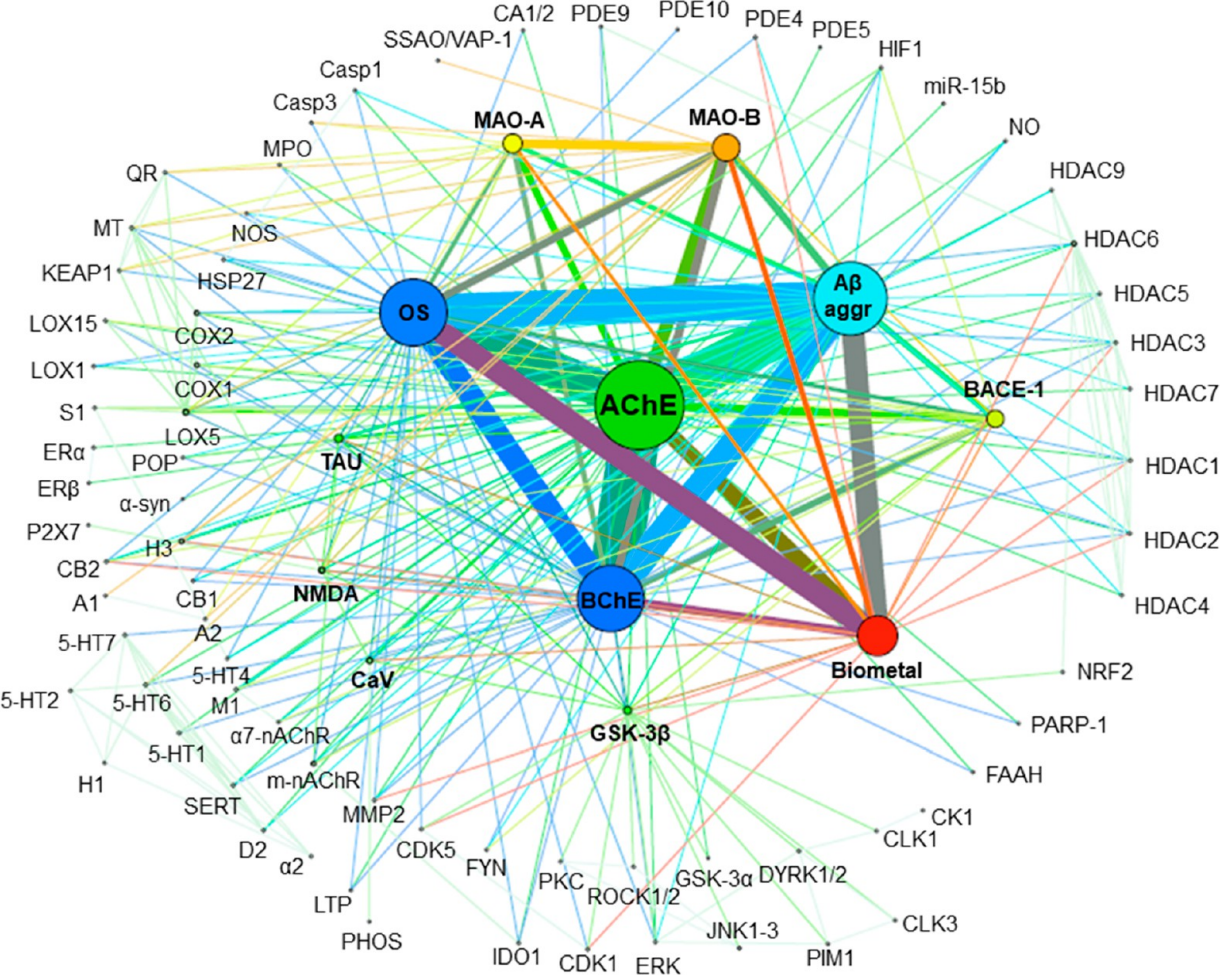
Mapping of the target combinations hit by multitarget anti-AD compounds developed in 1990–2020. Targets and binary target combinations appear as nodes and edges connecting the nodes, respectively. The size of the nodes and the thickness of the edges are proportional to the frequency with which each target or binary target combination has been pursued, respectively.

**Figure 15:**
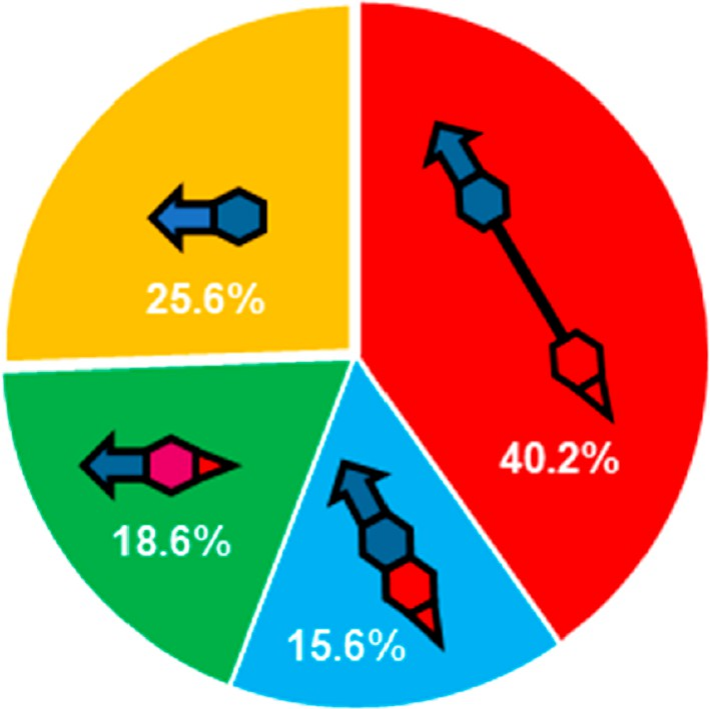
Design strategies for the multitarget anti-AD compounds developed in 1990–2020. The rate of use of privileged structures, i.e., compounds with inherent multitarget activity, is also shown (orange portion).

The presence of (hetero)aromatic rings is highly prevalent in small-molecule drugs,^50^ with the average number of aromatic rings in oral drugs being 1.6.^51^ Thus, most pharmacophores that are used to build multitarget agents feature at least one (hetero)aromatic ring, which is a favorable structural requirement for AChE PAS (or CAS) binding. This facilitates the design of hybrids that are at the same time multisite AChE inhibitors and multitarget agents able to hit other targets. Indeed, in a recent bibliometric analysis we found that (i) roughly three-quarters of the multitarget anti-AD compounds developed so far hit AChE as one of the targets (Figure 14) and (ii) linked hybrids are the preferred design strategy, accounting for 40% of all multitarget anti-AD compounds (Figure 15).^52^ This proportion is likely higher in the specific case of AChE-inhibitor-based multitarget compounds since linked hybrids tend to be rather large molecules that are particularly suitable to span the long cavities with multiple binding sites of proteins. However, the size of these compounds is perceived as a potential issue because, according to the Lipinski rules of five (Ro5), their use as drugs might be hampered by poor oral bioavailability.^53^

**Figure 16:**
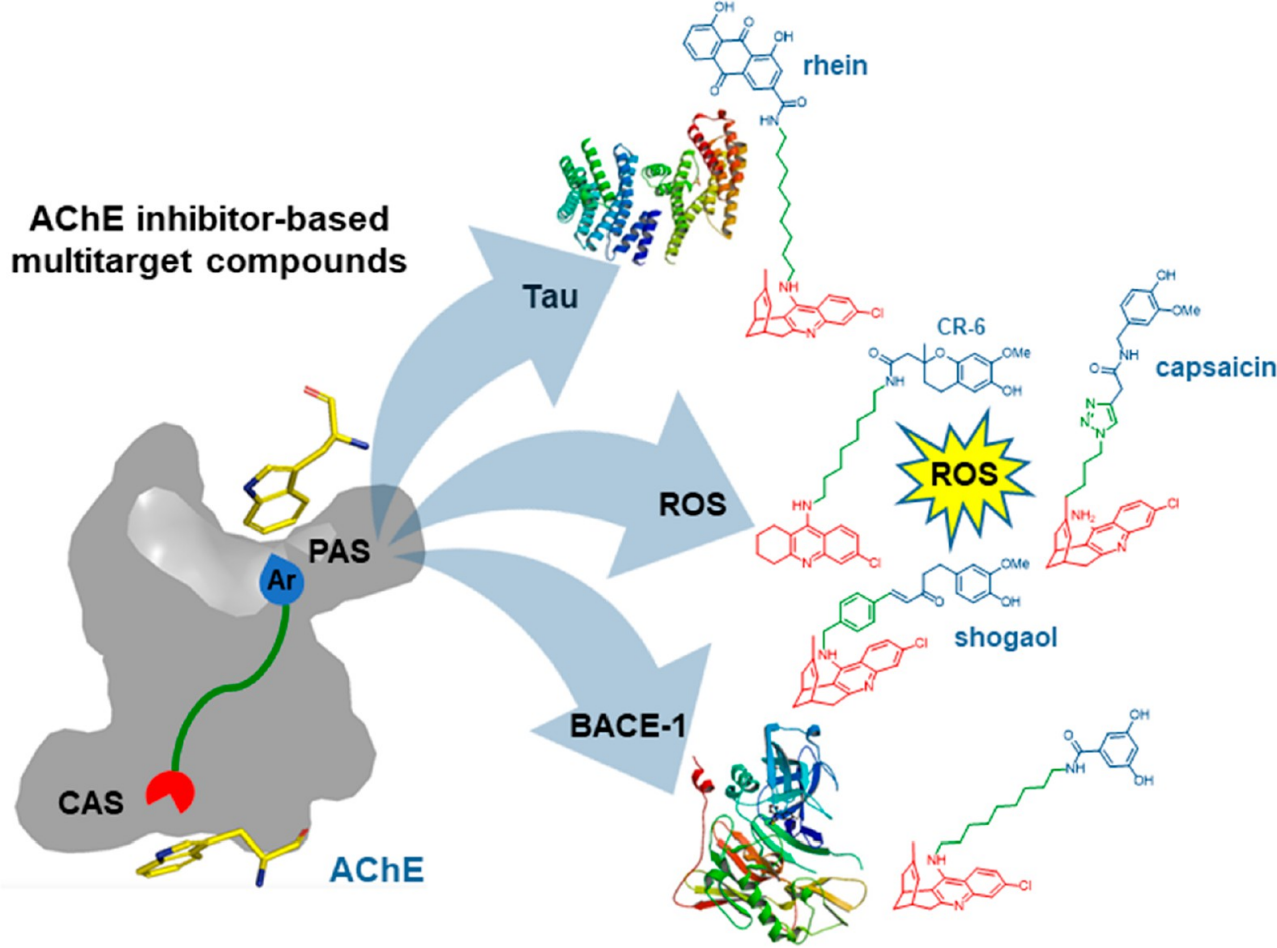
Multisite AChE-inhibitor-based multitarget agents.

We have developed several classes of multitarget linked hybrids that combine huprine or 6-chlorotacrine with polyphenol moieties, such as capsaicin,^54^ the chroman derivative CR-6,^41^ and shogaols,^45^ with the tau aggregation inhibitor rhein^33^ and/or with ligands of the aforementioned secondary binding pocket of BACE-1^42^ to simultaneously cope with the central cholinergic deficit and the oxidative stress or tau pathologies characteristic of AD (Figure 16). These compounds have demonstrated multiple activities in vitro, and despite being out of the Ro5, their chronic administration to different mouse models of AD (APP/PS1 and SAMP8) led to beneficial effects on cognition and to a reduction of oxidative stress, amyloid and tau pathology, neuroinflammation, and synaptic dysfunction.^33,41,54^ While this indirectly demonstrates that the compounds were able to cross the blood–brain barrier, biodistribution studies followed by HPLC/MS/MS determinations demonstrated that one of these hybrids reached the brain of C57BL6 mice even at a higher concentration than the anti-AD reference drug donepezil.^54^

**Figure 17:**
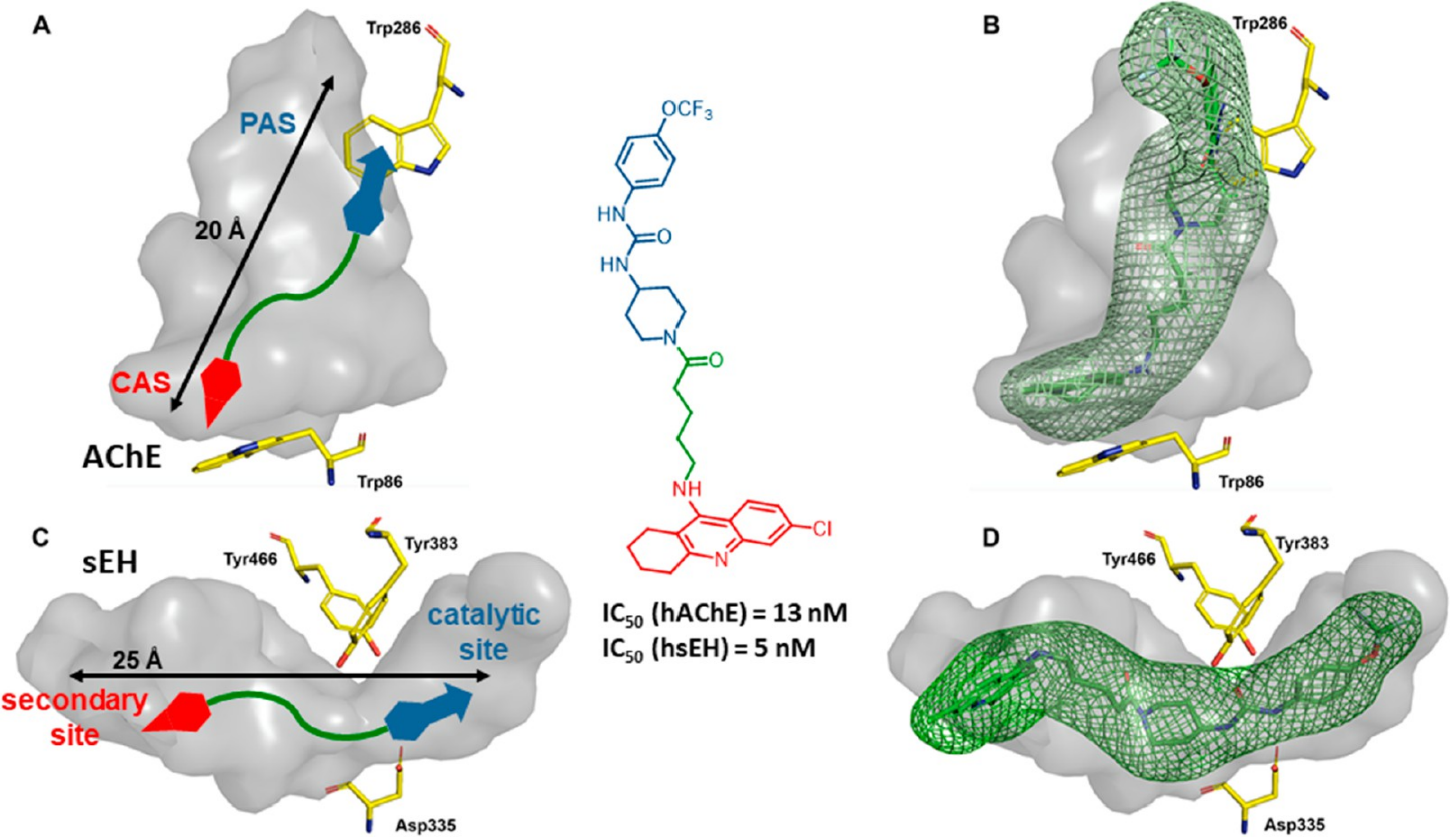
Design strategy for full occupancy of the active site cavities of hAChE (A) and hsEH (C). Placement of the lead TPPU–chlorotacrine hybrid within hAChE (B) and hsEH (D), based on MD simulations.

An example of multisite AChE-inhibitor-based multitarget agents is the recently developed first class of dual inhibitors of AChE and soluble epoxide hydrolase (sEH).^1^ ACh, upon activation of muscarinic M1 receptors, can promote the CYP-mediated metabolism of arachidonic acid to form anti-inflammatory epoxyeixocatrienoic acids (EETs). As EETs are degraded by sEH, dual inhibition of AChE and sEH in CNS should result in a sequential increase in EETs levels via ACh-mediated increased synthesis plus decreased degradation and hence in a potentiation of their antineuroinflammatory effects, apart from compensation of the cholinergic deficit. With this rationale and considering that, like AChE, sEH contains an elongated cavity (Figure 17A,C), we designed linked hybrids that combined 6-chlorotacrine or huprine Y and the reference sEH inhibitor TPPU. The whole occupancy of the active site cavities of both enzymes by the hybrids, as suggested by MD simulations, likely accounts for their in vitro single-digit nanomolar potency at both targets (Figure 17B,D). The lead compound showed favorable DMPK properties, a lack of neurotoxicity, and beneficial in vivo effects, rescuing memory, synaptic plasticity, and neuroinflammation in a mouse model of late-onset AD (SAMP8), after chronic oral administration of a low dose of 2 mg/kg.

## 5 Summary and Outlook

The particular architecture and amino acid composition of the AChE gorge, with a deep and narrow cavity, far from hampering its physiological functioning, make AChE highly efficient machinery to hydrolyze ACh. In diseases that occur with a cholinergic deficit, such as AD, the AChE gorge offers plenty of opportunities to design drug candidates that block that machinery and ameliorate cholinergic neurotransmission.

In this Account, we have shown different molecular hybridization approaches to gain occupancy of the AChE gorge and hence affinity. In a stepwise manner, we first extended the binding at the CAS of initial simple models and then designed multisite inhibitors by growing molecules to reach the PAS, or alternatively, starting from optimized PAS binders, the design was made to reach the CAS, with potency increases from several hundreds to more than 1.5 million-fold. Multisite AChE inhibitors often possess structural attributes or can be purposely designed to modulate other targets with large cavities, such as BACE-1 and sEH, or the aggregation of distinct amyloidogenic proteins.

Using these strategies, we have developed different structural classes with AChE inhibitory potencies up to the picomolar range, significant activity against other key targets in AD and other diseases, and beneficial effects in several AD mouse models. However, despite these impressive results, some challenges should still be addressed before these compounds can progress to regulatory preclinical and clinical development. Despite the power of molecular hybridization to increase potency, hybrids targeting proteins with deep cavities tend to be large molecules, potentially compromising DMPK properties and the interest of pharmaceutical companies. Like these hybrids, proteolysis-targeting chimeras (PROTACs) are large molecules containing two pharmacophores and a linker and are orally bioavailable and efficacious in vivo, despite being often out of the Ro5. The massive irruption of PROTACs in the drug discovery landscape, with a clear endorsement by pharmaceutical companies, should facilitate a better acceptance of multisite/multitarget AChE inhibitor-based hybrids. Meanwhile, other ways to exploit the vast territory of the AChE gorge toward new drug candidates will likely be uncovered.
